# Zidovudine Exposure in HIV-1 Infected Tanzanian Women Increases Mitochondrial DNA Levels in Placenta and Umbilical Cords

**DOI:** 10.1371/journal.pone.0041637

**Published:** 2012-07-27

**Authors:** Andrea Kunz, Nicole von Wurmb-Schwark, Julius Sewangi, Judith Ziske, Inga Lau, Paulina Mbezi, Stefanie Theuring, Andrea Hauser, Festo Dugange, Angela Katerna, Gundel Harms

**Affiliations:** 1 Institute of Tropical Medicine and International Health, Charité-Universitätsmedizin Berlin, Berlin, Germany; 2 Institute of Legal Medicine, Christian-Albrechts-Universität Kiel, Kiel, Germany; 3 Regional AIDS Control Program Mbeya Region, Ministry of Health and Social Welfare, Mbeya, Tanzania; 4 PMTCT Program Mbeya Region, Ministry of Health and Social Welfare, Mbeya, Tanzania; 5 Center for HIV and Retrovirology, Robert Koch Institut, Berlin, Germany; 6 Kyela District Hospital, Ministry of Health and Social Welfare, Kyela District, Tanzania; University of California, San Francisco, United States of America

## Abstract

**Background:**

Zidovudine (AZT) constitutes part of the recommended regimens for prevention and treatment of HIV-1 infection. At the same time, AZT as well as HIV-1 infection itself may induce mitochondrial damage. In this study, we analyzed the impact of prenatal AZT-exposure on mitochondrial alterations in HIV-infected women and their infants.

**Methods:**

Mitochondrial DNA (mtDNA) levels in placentas of HIV-1 infected Tanzanian women with and without prenatal AZT exposure, and in the umbilical cords of their AZT-exposed/unexposed infants were quantified using real-time PCR. Furthermore, we checked for the most common mitochondrial deletion in humans, the 4977 base pair deletion (dmtDNA4977) as a marker for mitochondrial stress.

**Results:**

83 women fulfilled the inclusion criteria. 30 women had been treated with AZT (median duration 56 days; IQR 43–70 days) while 53 women had not taken AZT during pregnancy. Baseline maternal characteristics in the two groups were similar. The median mtDNA levels in placentas and umbilical cords of women (311 copies/cell) and infants (190 copies/cell) exposed to AZT were significantly higher than in AZT-unexposed women (187 copies/cell; p = 0.021) and infants (127 copies/cell; p = 0.037). The dmtDNA4977 was found in placentas of one woman of each group and in 3 umbilical cords of AZT-unexposed infants but not in umbilical cords of AZT-exposed infants.

**Conclusions:**

Antenatal AZT intake did not increase the risk for the common mitochondrial deletion dmtDNA4977. Our data suggests that AZT exposure elevates mtDNA levels in placentas and umbilical cords possibly by positively influencing the course of maternal HIV-1 infection.

## Introduction

HIV-positive pregnant women can decrease the risk for in-utero vertical HIV transmission by intake of antiretroviral drugs (ARVs). Zidovudine (AZT) during pregnancy is a frequently used and WHO- recommended drug regimen [1]. However, it has been proven in human and animal studies that Nucleoside Reverse Transcriptase Inhibitors (NRTIs) like AZT can cause mitochondrial damages including depletion of mitochondrial DNA (mtDNA) [2]–[10].

One underlying mechanism of AZT-induced mitochondrial toxicity is the inhibition of human DNA polymerase gamma [11]–[12], the enzyme needed for replication of mtDNA. Other assumed mechanisms include increased mitochondrial oxidative stress, introduction of mtDNA mutations, negative effects on nucleotide phosphorylation and mitochondrial gene expression, depletion of L-carnitine and inhibition of the mitochondrial bioenergetic machinery [13]–[17].

However, also HIV-1 infection itself causes mitochondrial damage, like depletion of mtDNA and decreased activities of the mitochondrial respiratory chain complexes [18]–[21]. HIV-1 has been shown to induce mitochondrial toxicity in several ways: by loss of mitochondrial membrane potential, by increase of reactive oxygen species and through different mechanisms of the viral proteins Vpr, Tat and HIV protease [22].

In humans, the mitochondrial toxicity of antenatal NRTI-exposure was determined by measuring different mitochondrial parameters like the emergence of clinical mitochondriopathy or death [23]–[25], quantification of mtDNA [18], [26]–[29], analysis of mtDNA mutations [30] or expression of mitochondrial respiratory chain proteins [27].

Studies indicating NRTI-induced mitochondrial toxicity include a detailed analysis by Barret [23], who found a higher incidence of neuro-mitochondrial diseases in NRTI-exposed infants compared to NRTI-unexposed infants; Divi [28] found a decrease of mtDNA in umbilical cords of infants of HIV-positive mothers exposed to Combivir compared to infants of HIV-negative women. Shiramizu [26] measured lower mtDNA contents in placenta and cord blood of HIV-positive women following NRTI-exposure in comparison to HIV-negative individuals. Torres [30] detected a higher frequency of mtDNA mutations in umbilical cords of HIV-positive AZT exposed infants compared to HIV-negative infants.

In contrast, McComsey [27] identified increased mtDNA levels without changes in expression of mitochondrial respiratory chain proteins in infants of HIV-positive mothers having taken NRTIs compared to NRTI-unexposed infants of HIV-negative mothers. Williams [31] did not detect lower mental or motor functioning scores in HIV-exposed, uninfected infants who were in-utero exposed to ARVs including NRTIs compared to those unexposed to ARVs during pregnancy. Accordingly, two large cohort studies did not discover an increased risk for death or clinical manifestations suggestive of mitochondrial abnormalities in NRTI-exposed infants [24]–[25].

The only two studies comparing mtDNA levels exclusively among HIV-positive mothers and their infants came to contradictory conclusions. In blood samples of HIV-positive mothers and infants with and without prenatal AZT exposure, Poirier [29] found lower mtDNA levels in AZT-exposed infants, whereas Aldrovandi [18] identified higher mtDNA levels in women and newborns with antenatal AZT exposure.

Altogether, it has not been clarified whether the net effect of short-course AZT for drug-naive HIV-1 infected pregnant women and their infants is a positive or a negative one with regard to mitochondriopathy. In the present study, we therefore quantified the mtDNA content in placentas of HIV-1 positive women with and without antenatal AZT exposure and in umbilical cords of their AZT exposed/unexposed infants. Furthermore, we checked for the most common mitochondrial deletion in humans, the 4977 base pair deletion (dmtDNA4977) as a marker for mitochondrial stress [32].

## Methods

### Ethics Statement

Ethical approval was obtained from the local Mbeya Medical Research and Ethics Committee, the National Institute for Medical Research of Tanzania and the ethical committee of Charitè-Universitätsmedizin Berlin, Germany. All participants had given written informed consent, and data and samples were treated strictly confidentially.

### Clinical Samples

The present study is a sub-evaluation of an observational study analyzing feasibility and adherence regarding combination prophylaxis for the prevention of mother-to-child transmission of HIV-1 (PMTCT) at Kyela District Hospital (KDH), Mbeya Region, Tanzania between October 2008 and September 2009 [33].

According to the WHO guidelines from 2006 and the National Tanzanian PMTCT guidelines from 2007 [34]–[35], HIV-1 positive women without treatment indication (CD4 cell count >200 cells/µl) took AZT, starting in gestational week 28 (2×300 mg per day), or anytime thereafter followed by single-dosed Nevirapine (200 mg) at labor onset and AZT (300 mg) every three hours, plus Lamivudine (150 mg) every 12 hours during labor; additionally, the mother took postnatal AZT/Lamivudine for one week. Newborns received single-dosed Nevirapine after birth and AZT for 1 week [33].

PMTCT clients who had taken antenatal AZT for at least 4 weeks and who delivered at KDH within the study period were eligible for this sub-study if placenta and/or umbilical cord specimens were available, if the mother delivered a singleton, and if both mother and infant were alive 48 hours after birth. The same eligibility criteria applied for HIV-1 positive women delivering at KDH who had not taken AZT during pregnancy, constituting the control group. These women were offered the same intrapartum and postpartum drug regimen as described above. Since the half-life of mtDNA in mammalian cells is several days [36], ARV exposure during labor and thus shortly before the samples were taken should not affect mtDNA levels and the presence of the dmtDNA4977 deletion. Aliquots of placenta and umbilical cords of HIV-1 positive women and their infants were sampled at delivery, frozen and stored at −20°C for future DNA extraction. For the detection and quantification of HIV-1 RNA viral load in newborns, a plasma sample from delivery was used and analyzed by RT-PCR according to our previously published protocol [37]. Socio-demographic data, AZT intake and maternal and newborn parameters were documented using specific questionnaires during antenatal care and at delivery [33].

### Quantification of Mitochondrial DNA and the Mitochondrial 4977 bp Deletion (dmtDNA4977)

#### DNA extraction

50 mg of each tissue (placenta and umbilical cord) were subjected to DNA extraction using the Invisorb Spin tissue mini kit. Samples were incubated at 52°C overnight and vortexed in an Invisorb Gyrator to intensify the lysis process (both STRATEC Molecular, former Invitek, Berlin, Germany). DNA was eluted in 55 µl elution buffer and stored at 4°C or analyzed immediately by PCR.

#### Real-time PCR for absolute quantification of nuclear DNA

Nuclear DNA was quantified as previously described [38]. Briefly, a 98 bp fragment of the telomerase gene (forward primer: 5′-GGC ACA CGT GGC TTT TCG-3′, reverse primer: 5′-GGT GAA CCT CGT AAG TTT ATG CAA-3′) was used to specifically amplify nuclear DNA. Dilutions of control DNA (Promega, Mannheim, Germany) were prepared (100 ng, 10 ng, 2.5 ng, 1.0 ng, 0.5 ng, 0.1 per µl) and used as standards assuming approximately 1,500 haploid copies of the telomerase gene in 10 ng total control DNA [39]. Every sample was amplified in triplets using 2 µl of pure DNA extract. The thermal cycling program was: 10 s at 50°C and 10 min at 95°C for enzyme activation (allowing an automated hot start PCR), 40 cycles of denaturation for 30 s at 94°C and 60 s annealing at 65°C on an ABI 7300 Real Time PCR System (Applied Biosystems). This approach allowed calculation of the amount of DNA/µl in ng and the number of cells/µl.

#### Real-time PCR for absolute quantification of mitochondrial DNA

Absolute quantification of dmtDNA4977 and total mtDNA was done as presented in [40] with some changes. To test the precision and reliability of the real time PCR, the primer pair L15/H16 (np: L3304-L3328/H3564-H3539 in [41] was employed to produce synthetic ND1 targets of 260 bp in length, which were used as specific template molecules for undeleted wildtype mtDNA in the subsequent PCRs. These ND1 targets were purified from primer sequences by ultrafiltration through Centricon 30 membranes and quantified after gel electrophoresis and detection on a gel imaging system (Geldoc, Biorad). Then, the molecules were serially ten-fold diluted from 10^6^ to 1 copies/10 µl, and mixed with 100 ng mouse DNA (15,000 haploid genome equivalents) per 10 µl each to simulate the complexity of the human genome.

For quantification of dmtDNA4977, a 238-bp fragment was amplified using the primer pair L35/H45 (np: L8285-L8310/H13499-H13475, [41]). These primers flank the breakpoints of the common deletion and preferentially amplify dmtDNA4977 under short cycle conditions. Known numbers of the purified 238-bp products served as targets for quantification of specifically deleted molecules in our DNA samples. The standard preparation was done in the same way as described for total mtDNA. For detection of specific PCR products, we used FAM-labeled probes for wildtype and VIC-labeled probes for dmtDNA4977. For sample analysis, a real time duplex-PCR was performed using standard mixtures of 1, 10, 100 and 1000 dmtDNA4977 specific fragments on a background of 106 wildtype specific template molecules. Each amplification was done in triplets. PCR was performed using a standard Immuno buffer and an Immolase polymerase at a concentration of 1 U/25 µl per reaction (both Bioline Germany). The concentrations of the primers, magnesium chloride, and dNTPs were 0.0002 mM, 1.5 mM, and 0.2 mM per dNTP, respectively. Dimethylsulfooxide (DMSO, SIGMA, Steinheim, Germany) was added as an additive in a concentration of 2%.

### Statistical Analysis

For statistical analysis, the non-parametric Mann-Whitney U test was used to assess significant differences with regard to continuous variables between two independent samples whereas the Chi-Square test or Fisher’s exact test were applied to analyze the independence of categorical variables. Testing of significant correlations between two continuous variables was done by Pearson’s correlation coefficient. Levels of mtDNA in placenta and umbilical cord were logarithmically transformed and presented as box and whisker plots. For descriptive analysis, median and interquartile ranges (IQR) were calculated. Two-sided tests were used and P<0.05 was considered as statistically significant. Statistical analysis was carried out using PASW Statistics 18 (SPSS Inc., Chicago, Illinois, USA).

## Results

### Sample Characteristics

In total, 83 women and their infants fulfilled the inclusion criteria. Samples of 30 women having taken AZT antenatally for a median duration of 56 days (IQR 43–70 days) and their infants were compared with samples of 53 women without pre-delivery AZT exposure and their infants. No significant differences between the two groups could be observed with regard to maternal CD4 cell count or socio-demographic variables like age, weight, education or marital status of the mother or infant’s birth weight and length (table 1).

**Table 1:** Demographic and clinical characteristics of HIV-1 infected women and their infants with or without AZT exposure during pregnancy.

		No antenatal AZT (n = 53)		Antenatal AZT (n = 30)	
Characteristics	n	% or median (IQR)	n	% or median (IQR)	p-value
Duration of AZT intake, days	53	No intake	30	56 (43–70)	
Maternal age, years	51	25 (23–29)	30	28 (25–30)	0.12
Maternal weight, kg	46	59 (55–68)	29	61 (56–65)	0.38
Education, years	45	7 (6–7)	26	7 (7–7)	0.26
Marital status, married	50	72	28	75	0.77
Gravidity	51	3 (2–3)	30	3 (2–3.3)	0.54
Parity	51	2 (1–2)	30	2 (1–2.3)	0.68
Maternal CD4 count at delivery, uL	36	307 (184–462)	19	402 (272–492)	0.10
Prematurity (<37 wk gestation)	51	3.9	30	13.3	0.19
Mode of delivery, caesarean section	49	6.1	30	6.7	1.0
Apgar score at 1 minute	41	9 (8–9)	29	9 (8.5–9)	0.08
Female sex of infant	50	58	30	40	0.12
Infant birth weight, g	48	3100 (2800–3300)	29	3200 (2950–3500)	0.30
Child length, cm	42	48 (46–50)	27	48 (46–50)	0.71
Child head circumference, cm	42	35 (34–36)	27	36 (34–36)	0.11

The proportion of HIV-1 infected newborns at birth was similar in the two groups; 4/44 AZT-unexposed infants versus 2/30 AZT-exposed infants (p = 1.0). Since the exclusion of data of HIV-1 infected infants did not change the results (data not shown) we decided to keep the data of HIV-infected infants in the analysis.

### Levels of mtDNA in Placenta and Umbilical Cord

**Figure 1:**
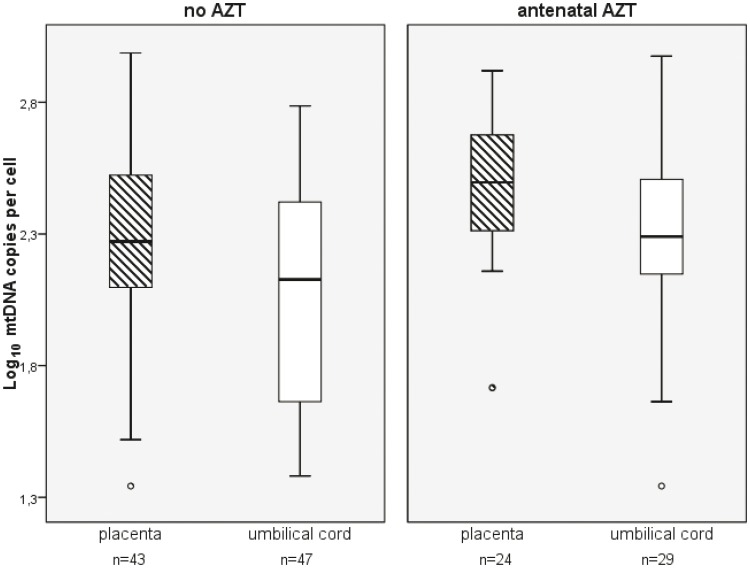
Box/whisker plots of mitochondrial DNA levels in AZT-exposed and AZT-unexposed placenta and umbilical cords. Box and whisker plots show mitochondrial DNA (mtDNA) levels in placentas of HIV-1 infected women and in umbilical cords of their infants according to exposure to antenatal AZT. The median mtDNA level was significantly higher in women exposed to AZT compared to women without AZT-exposure. Accordingly, the median mtDNA level was significantly higher in umbilical cords of infants exposed to AZT compared to infants without AZT-exposure.

The median mtDNA level was significantly higher in women exposed to AZT (311 copies per cell, IQR 166–475) compared to women without AZT-exposure (187 copies per cell, IQR 115–352; p = 0.021). Accordingly, the median mtDNA level was significantly higher in umbilical cords of infants exposed to AZT (190 copies per cell, IQR 121–323) compared to infants without AZT-exposure (127 copies per cell, IQR 70–234; p = 0.037). The box and whisker plots of mtDNA levels in placentas of HIV-1 infected women and in umbilical cords of their infants according to antenatal AZT-exposure are shown in figure 1. Restricting the analysis to women having taken AZT during pregnancy, we did not find a correlation between the duration of antenatal AZT intake in days and mtDNA levels in placenta (p = 0.95) and umbilical cord (p = 0.76).

### Frequency of dmtDNA4977 in Placenta and Umbilical Cord

The mitochondrial 4977-bp deletion was rarely found in placental tissues (1/43 AZT- unexposed women versus 1/24 AZT-exposed women, p = 1.0). These two women displayed the mtDNA4977 at very low proportions with mutant DNA in percentage of the total DNA being 2.6E-05 (woman unexposed to AZT and 2.9E-05 (woman exposed to AZT).

In umbilical cords of infants, the dmtDNA4977 was detectable in 3/47 (6.4%) infants without AZT exposure and in no infant with prenatal AZT exposure (0/23); this was not statistically different (p = 0.55). The proportion of mutant DNA in percentage was higher in umbilical cords than in the placental tissues: 9.4E-04, 6.9E-03 and 1.4E-02.

In no case, the dmtDNA4977 was detected in both placenta and umbilical cord of a mother-child-pair.

## Discussion

There is a high level of evidence from animal and human studies that both AZT and HIV-1 can cause mitochondrial toxicity and reduce mtDNA levels [2]–[10], [18]–[22].

As a main finding, our study detected higher mtDNA levels in AZT-exposed mother-child- pairs compared to unexposed ones. This is in accordance with findings by Aldrovandi [18], who analyzed samples of HIV-positive mothers with similar HIV-1 disease progression but differing antenatal exposure towards NRTIs; higher mtDNA levels in the AZT-exposed mother-infant group were detected even five years later. Contradictingly, Poirier et al. [29] achieved results indicating lower mtDNA levels in the AZT-exposed infants. Yet, this study is only partly comparable because both infant groups differed remarkably in the HIV-1 disease progression of their mothers, with those having taken AZT displaying a tenfold higher viral load and >twofold lower CD4 cell count levels [29].

We analyzed mtDNA levels in placentas and umbilical cords instead of PBMCs. While mtDNA levels in PBMCs do not necessarily correlate with mtDNA levels in other tissues or with clinical signs of mitochondriopathy [42]–[44], placentas and umbilical cords seem to reflect mitochondrial changes induced by AZT: antenatal AZT–exposure led to decreased mtDNA levels in placentas and umbilical cords of fetal patas monkeys, which was correlated with an increase in mitochondrial morphological changes [6], [9]. This suggests that mtDNA levels in placenta and umbilical cord reflect mitochondrial damage and are thus suitable markers of potential mitochondrial toxicity following AZT-exposure.

To our knowledge, this study is the first to analyze the presence and the relative amount of dmtDNA4977, the most common mitochondrial mutation, in placentas of HIV-infected women and umbilical cords of their infants with and without exposure to ARVs. In our study, dmtDNA4977 detection was rare and could only be detected in placentas of a single AZT-exposed and AZT-unexposed woman each, at very low proportions. This finding is consistent with other studies [45]–[46] which did not find the dmtDNA4977 in human placenta samples.

The occurence of mtDNA4977 has been shown to be age-dependent and was undetectable in tissue biopsies of children [47]–[48]. However, the deletion has been identified in brain, liver, kidney, heart, and muscle samples taken at autopsy of deceased neonates [49]. The authors speculate that mtDNA4977 could be generated by perinatal hypoxia or temporary oxygen oversaturations during the intensive care of the neonates.

Here, we demonstrate that mtDNA4977 can also be observed in newborns of HIV-1 infected women. We identified the dmtDNA4977 in umbilical veins of 3/70 (4.3%) infants, all unexposed to AZT. The amount of dmtDNA4977 out of the total mitochondrial DNA was low and varied between 0.00094% and 0.014%. As there was no significant difference between AZT-exposed and AZT-unexposed newborns (p = 0.55), we suggest that our findings could be explained by the influence of external factors other than AZT. It is well known that mitochondrial mutagenesis depends on many different factors such as alcohol [38], [50] or nicotine [51]. While these stressors are known to damage mitochondrial DNA there are also findings on protective factors, e. g. green tea [52] or other dietary components. We did, however, not investigate such possibly influencing factors. It is also imaginable that the difference was not significant due to low sample size. On the other hand, since none of the AZT-exposed infants displayed the deletion, we cannot rule out the possibility that AZT has a benefical impact with regard to the emergence of the mtDNA4977.

Altogether, we did not find evidence for an increased risk resulting from AZT for the most common mitochondrial deletion in placenta or umbilical cord tissues of HIV-1 infected women and their infants.

There is no doubt that AZT can cause mitochondrial toxicity. However, the mitochondrial toxicity of AZT may be counterbalanced by the positive effect of AZT on maternal HIV-1 infection. Compared to individuals under ARV long-term treatment, the situation may be different in pregnant, drug-naive women taking AZT for a short period only. Generally, HIV-infection of the mother has a profound derogatory effect on the cell-mediated immunity and T-cell maturation of the infant [53]. In HIV-positive individuals, the inflammatory cytokine tumor necrosis factor alpha (TNF-alpha) is elevated [54]–[55] which also applies to placental trophoblastic cells [56]–[57]. However, increased TNF-alpha levels lead to mitochondrial DNA damage including mtDNA depletion [58]–[60]. Interestingly, AZT has been shown to down-regulate the expression of TNF-alpha in placental tissue [61]; this mechanism could prevent HIV-induced mitochondrial damage and explain the higher mtDNA levels in placenta and umbilical cord samples of AZT-exposed women and infants as observed in our study.

Accordingly, it has been shown that mtDNA levels in PBMCs of HIV-positive adults and infants increase after start of ARV treatment. This finding has been interpreted by some authors as restorative effect due to suppression of HIV-1 infection and by others as over-replication to compensate for mitochondrial dysfunction [21], [44], [62]. However, we believe that mtDNA over-replication as a sign for mitochondrial dysfunction is unlikely in our study, as it has been shown that antenatal AZT exposure leads to decreased mtDNA levels but increased mitochondrial morphological damage in placenta and umbilical cords of fetal patas monkeys [6], [9].

There is a wide variety of factors influencing the mitochondrial toxicity of NRTIs, like the type of NRTI (e.g. d4T or AZT), the material to be analyzed (e.g PBMCs or tissues), mitochondrial outcome parameter (e.g mtDNA or activities of mitochondrial respiratory chain complexes) or stage of HIV-1 disease; this could explain the somewhat ambiguous results of the studies conducted so far.

There are also technical issues involved. Contamination with platelets is one possible confounder of measuring mtDNA content in PBMCs by real-time PCR, as platelets are not completely removed during standard Ficoll gradient separation method [63]–[64]. In real time PCR, the mtDNA/nDNA-ratio is calculated; as platelets do contain mtDNA, but not nDNA, every increase of platelets leads automatically to a higher mtDNA/nDNA-ratio resulting in higher calculated mtDNA levels.

Furthermore, other factors like human mitochondrial DNA mutations and haplotypes may influence the development of HIV- and NRTI-associated mitochondrial dysfunction [65]–[70]. There are nine known European mitochondrial DNA haplotypes, whereas the greatest and still insufficiently characterized variety of mitochondrial haplotypes can be found in Africa; one study revealed 105 haplotypes with 75 forming a single, unique African haplogroup L [71]. Since we analyzed samples from Tanzanian women, it cannot be excluded that other populations harboring different mitochondrial DNA haplotypes react differently towards AZT-exposure.

In conclusion, in our setting of relatively immunocompromized drug-naive pregnant women from rural Tanzania, antenatal AZT intake seemed to improve mitochondrial parameters in the women and their infants.
